# Insulin Release Mechanism Modulated by Toxins Isolated from Animal Venoms: From Basic Research to Drug Development Prospects

**DOI:** 10.3390/molecules24101846

**Published:** 2019-05-14

**Authors:** Beatriz Elena Sarmiento, Luis Felipe Santos Menezes, Elisabeth F. Schwartz

**Affiliations:** Departamento de Ciências Fisiológicas, Instituto de Ciências Biológicas, Universidade de Brasília, Brasília, DF 70910-900, Brazil; beatrizelenasarmiento@gmail.com (B.E.S.); luisfelipe_100@outlook.com (L.F.S.M.)

**Keywords:** diabetes, insulin release, ion channel, natural products, toxins

## Abstract

Venom from mammals, amphibians, snakes, arachnids, sea anemones and insects provides diverse sources of peptides with different potential medical applications. Several of these peptides have already been converted into drugs and some are still in the clinical phase. Diabetes type 2 is one of the diseases with the highest mortality rate worldwide, requiring specific attention. Diverse drugs are available (e.g., Sulfonylureas) for effective treatment, but with several adverse secondary effects, most of them related to the low specificity of these compounds to the target. In this context, the search for specific and high-affinity compounds for the management of this metabolic disease is growing. Toxins isolated from animal venom have high specificity and affinity for different molecular targets, of which the most important are ion channels. This review will present an overview about the electrical activity of the ion channels present in pancreatic β cells that are involved in the insulin secretion process, in addition to the diversity of peptides that can interact and modulate the electrical activity of pancreatic β cells. The importance of prospecting bioactive peptides for therapeutic use is also reinforced.

## 1. Introduction

Diabetes is a disease caused by a progressive and chronic metabolic malfunction, characterized by the body’s inability to maintain the glucose homeostasis condition [1]. Individuals with this disease may develop hyperglycemia, hyperinsulinemia, and hypertriglyceridemia [2]. Among the possible causes there are ethnicity, age group, obesity, sedentarism, family history [1,3] and also diseases, for example cystic fibrosis or pancreatitis, in addition to drugs such as glucocorticoids or the effects of organ transplantation [4]. There are four types of diabetes: type 1 diabetes (T1D), type 2 diabetes (T2D), gestational diabetes mellitus and specific types of diabetes that are caused by other reasons, such as monogenic diabetic syndrome, pancreatic or drug-related diseases, and chemical inducers [5]. In the United States, 30 million people are now afflicted with diabetes, 84 million are in prediabetes condition, and this disease was the seventh leading cause of death in 2015 [6]. Currently, there are about 422 million cases of diabetes in the world, with estimated growth to 592 million in 2035; of these, T2D is the most prevalent, responsible for about 95% of cases recorded [7]. T2D can occur due to the progressive loss of β cells secreting insulin or its resistance to absorption [8], while T1D occurs due to the destruction of autoimmune β cells, thus leading to very little or no insulin production, and gestational diabetes is usually discovered in the second or third trimester of pregnancy, but its causes are not known [5]. 

In patients with T2D, frequent infections and difficulties in wound healing are commonly diagnosed, causing multiple damage and complications in the organism, thus contributing to a high rate of diabetes mortality [9]. For these reasons, dietary changes, exercise and the continuous monitoring of glycemic levels can aid in the prevention and treatment of diabetes [10,11]. Treatment agents like sulfonylureas, thiazolidinedione and glucagon-like peptide agonist 1 (GLP-1) help in the treatment of T2D, while exogenous insulin applications are required for T1D [12]. However, current treatment agents can cause several adverse effects [13], and therefore the treatment of this disease demands multiple medications to control blood glucose levels [14]. 

In recent years, several drugs have become available for use, and some even have demonstrated that they cause weight loss and reduce the cardiovascular risk [14], but due to several limitations in the actual treatment, the development of new drugs is still necessary. Peptides isolated from animal venoms could be a new path to follow, and because some of them have selectivity and affinity for their molecular targets, they could be used to study, correlate and possibly counteract the biological malfunction in the insulin release process. Based on the ability of many of these peptides to interact with ion channels, in this review we will focus on the main ion channels that are involved in the insulin secretion process, and on peptides isolated from animal venoms that modulate the insulin release with possible and actual therapeutic use in T2D treatment.

## 2. Insulin Release Mechanism

The endocrine function of the pancreas is to control blood glucose levels; this organ is comprised of exocrine (97%) and endocrine tissues (1–2%) [15,16]. Most of the endocrine cells in the islet of Langerhans are called β cells (50%), which are responsible for secreting insulin into the blood circulation in response to an increase in the plasma glucose levels, and they are the only source of insulin known in mammals [16]. Several nutrients stimulate insulin secretion from pancreatic β cells, and glucose, as the most potent and best stimulus for this response [16]. 

### Metabolic Events and Electrical Activity in β Cells

Cells in the islet of Langerhans are excitable [17], and high concentrations of glucose depolarize the membrane of pancreatic β cells [18]. The mechanism that links glucose sensing to insulin secretion (GSIS) has been extensively studied in β cells and involves the activity of metabolic events such as glycolysis [16,19], helped by glucose transporters (GLUT) like GLUT2 and Type-1 glucose transporters in rodents and humans, respectively [19,20,21,22]. Glucose enters the cells and is phosphorylated by glucokinase (GCK) to glucose-6 phosphate (G6P). In the cytoplasm, G6P enters the glycolytic pathway until pyruvic acid is obtained, which will produce Acetyl-CoA. This, in turn, starts the Krebs cycle and oxidative phosphorylation in the mitochondria, leading to a rise in the intracellular ATP/ADP ratio [16,19] to induce a cascade of electrochemical events that culminate in the increase of intracellular calcium concentration and insulin secretion [19].

Several ion channels are present in mammals [16,19,23,24,25], and in humans, the plasma glucose level in the fasting state is around 4–5 mM. In this physiological condition the β cells are electrically silent at their resting potential, which oscillates about −60 mV; however, when glucose is low or absent the resting potential is around −70 to −80 mV [16,19]. As a consequence of glucose metabolism, the ATP/ADP ratio increases, which leads to the closure of the ATP sensitive potassium channel (K_ATP_), promoting a slow depolarization of the membrane potential, which is also dependent on the activity of TRP channels [26]. Once depolarization of the membrane potential (Vm) begins and reaches around −40mV, the voltage gated sodium channels (Na_v_) open, and voltage-dependent calcium channels (Ca_v_) are activated, which allows the increase in Na^+^ entry into the cells and causes further depolarization [17]. At −20 mV, the Ca_v_ open, and the influx of Ca^+2^ ions increases the concentration of intracellular calcium and, therefore, insulin release by exocytosis [26,27].

When extracellular glucose increases from basal to high levels (>10 mM), the increment of insulin release in the first few minutes (1 to 8 min) is sharp and transient, and it is referred to as the first phase of insulin secretion. A second increase is observed when high glucose concentration is maintained, gradually increasing the rate of release to a plateau after an additional 25 to 30 min, although the intensity of secretion is slower than in the first phase, and it is sustained until reaching euglycemia. The behavior of insulin release is therefore biphasic [28,29,30,31], and insulin thus regulates the storage of nutrients and the glucose uptake in the adipose tissue and muscle, which is extremely important in the life of mammals [16].

The closure of the K_ATP_ channel has been associated with the first phase of insulin secretion, letting the depolarization and exocytosis of a small pool of granules take place [32,33,34,35]. In the second phase of insulin secretion, the mechanisms involved seem to be more complex. They are still controversial, but it is proposed that the mechanism that signaled this secretion is a K_ATP_ channel-independent pathway of glucose signaling [36,37,38]. Finally, voltage-dependent potassium channels (K_v_) and calcium sensitive voltage dependent potassium channels (KCa) repolarize the membrane potential and, in consequence, stop insulin secretion [39,40,41]. It is important to mention that the K_ATP_ channel-dependent pathway is referred to as the “triggering” pathway, and it is the main pathway of GSIS; it is also known that the increase of intracellular calcium by the K_ATP_ channel-independent pathway has a potential effect on the exocytotic machinery [17,42,43,44]. In this way, these two pathways are synergistic.

In this section, we outline how the different ion channels contribute to the electrical activity pattern and play an important role in the GSIS [19,45]. The electrical activity can be different among species and depends on the different expression of these ionic channels [17,19,46]. However, the aspects or mechanism of glucose sensing are the same [17]. 

## 3. ATP Sensitive Potassium (K_ATP_) Channels and Insulin Secretion

K_ATP_ channels in pancreatic β cells were initially described by Cook and Hales (1984), where the physiologic role has been mostly characterized [33,47] and recently reviewed elsewhere [26,48]. In β cells and neurons, the function and the structural arrangement of K_ATP_ channel are formed by K_IR_6.2 subunit/SUR1 receptor [49], which are associated in a 1:1 stoichiometry [50]. Here, the most important functional and electrophysiological characteristics related to insulin release will be presented. 

The role of the K_ATP_ channels is very important in the relationship between the energy metabolism of the cell and membrane excitability, because the activity of these channels in the presence of intracellular ATP is reduced; in contrast, it is enhanced by the presence of MgADP, leading these channels to have the status of metabolic sensor [51]. The SUR 1 receptor is responsible for nucleotide binding and hydrolysis of the nucleotide binding folds [19,52], and the K_IR_6.2 subunit is responsible for pore formation and ion selectivity [53]. Furthermore, the N and C terminals are intracellular in K_IR_6.2 channels and structurally important in the regulation by ATP and sulfonylureas [54]. The inhibition by ATP is the most predominant characteristic of K_ATP_ channels, where only one ATP molecule is enough to inhibit the channel and facilitate insulin secretion [55], but the ATP/ADP ratio is more relevant than ATP alone in regulating the K_ATP_ [47]. In this way, in the presence of ATP or ADP, the K_ATP_ channel regulation requires both subunits to work together and in a balance for the inhibitory effect of ATP and the stimulatory effect of ADP [19]. In addition, the ATPase activity of SUR1 drives discrete conformational changes that modulate the K_ATP_ channel gating [56]. 

From the point of view of electrical activity, the main function of K_ATP_ channels in β cells is to determine the resting potential in the membrane [57], and the electrophysiological properties of K_ATP_ channels have been thoroughly characterized [17,45,58,59]. It is important to mention that K_ATP_ channel closure alone is not sufficient to produce membrane depolarization; a depolarized membrane current is also required [60]. The increase of glucose levels from 6 to 20 mM [17] triggers depolarization, leading to a loss of K_ATP_ channel activity by >75%, an elevation of [Ca^+2^]_int_ and subsequent electrical activity [60,61] that induces a much stronger stimulation of insulin release [17]. Furthermore, these channels can be regulated by lipids such as long chain acyl-CoA esters that interact with the carboxyl terminal domain of K_IR_ 6.2 subunit [62]; they are the most potent stimulator of K_ATP_ channel activity in human pancreatic β cells [63]. Phosphatidylinositol-4, 5-biphosphate (PIP2), a second messenger that increases K_ATP_ channel activity [64], can regulate the K^+^ channel activity and therefore insulin release, because this ligand can interact with other proteins that also regulate K_ATP_ channels, and it has been suggested that these proteins could be involved with the exocytotic machinery [65]. 

For example, syntaxin-1A (Syn-1A) is a potent endogenous inhibitor of K_ATP_ [66] and regulates the K_ATP_ channel trafficking [67,68]. This is also the case of Leptin [69], which can activate different ion channels, including K_ATP_, both in central neurons and β cells [70,71,72]. Activation of G protein coupled receptors mediates parasympathetic input and autocrine feedback in pancreatic β cells, thus facilitating glucose-stimulated insulin secretion. This is due to reduced K_ATP_ channel activity as a response to the activation of P2Y receptors and muscarinic receptors in the β cell membrane, which lead to an increase in [Ca^+2^]_int_ and a decrease in PIP2 [73,74,75,76,77]. Protein phosphorylation is also a regulatory mechanism for K_ATP_ channel conductivity and density in pancreatic β cells, and protein kinase A (PKA) and protein kinase C (PKC) are responsible for it [78,79,80]. In the K_IR_6.2 subunit, PKA phosphorylation sites are threonine 224 and serine 372, and the phosphorylation of these residues significantly increases the probability of having an open channel [78]. Meanwhile, PKC phosphorylates the conservative threonine 180 and, as a result, decreases the K_ATP_ channel activity [80]. 

### Inhibitors of K_ATP_ Channel Activity

Inhibitors of K_ATP_ channel activity can act either by interacting with SUR receptors or with K_IR_6.2 subunits [81]. Sulfonylureas such as tolbutamide, gliclazide and glimepiride have a high affinity to SUR receptors [82,83,84] and close the channel, but these compounds can also interact with K_IR_6.2 subunits with low affinity [83,85]. On the other hand, there are other compounds such Imidazolines and antimalarials that block K_ATP_ channels by binding to the K_IR_6.2 subunit [86,87,88]. The antimalarial agents known as quinoline drugs induce a variety of side effects, and the most important are cardiac abnormalities, neuropsychiatric disturbances and hypoglycemia [89]. Gribble and co-workers (2000) showed that mefloquine, quinine, and chloroquine inhibited the K_IR_6.2/SUR1 in pancreatic β cells, and that inhibition is mediated by interaction of the drugs with the K_IR_6.2 subunits of the K_ATP_, rather than with the regulatory SUR subunit [88,90]. Lopez-Izquierdo and co-workers (2011) postulated that the inhibition of K_ATP_ channels by mefloquine results from the interaction between PIP_2_ and K_IR_ channels [91], a proposition which is supported by a number of studies that conclude that this interaction directly controls K_IR_ channels gating [92,93,94,95]. PNU99963 and PNU37883A are selective but structurally different non-sulfonylurea K_ATP_ channel inhibitors [96,97], and electrophysiological studies showed that PNU37883A at low micromolar concentrations inhibited K_ATP_ currents in single vascular smooth muscle [98]. Moreover, it was a direct pore blocker for the K_IR_6.1 subunit, while PNU99963 interacted with high nanomolar affinity with the sulfonylurea receptor [99,100].

## 4. Nav Channels and Insulin Secretion

Voltage dependent Na^+^ channels (VDSC) play a central role in cellular excitability [101], and their presence in mouse islets was described for the first time by Plant in 1988 [102]. VDSC are dually influenced by the membrane potential, because they are activated in response to membrane depolarization and inactivated as a consequence of sustained depolarization [103]. Pancreatic β cells have a voltage-dependent Na^+^ current that, owing to the voltage dependence of inactivation, is unlikely to play an important role in glucose-stimulated electrical activity [102]. In this scenario, at the resting potential most VDSC are inactivated or closed, and their inactivation can be removed only by membrane hyperpolarization [19]. Hiriart and Matteson (1988) demonstrated for the first time that all rat β cells contain VDSC and also indicated that this current is functionally important for stimulus secretion coupling, because TTX (Na^+^ channel blocker) partially inhibits glucose-induced insulin secretion, but at higher glucose concentrations TTX clearly inhibited the secretory response [104]. Later, it was demonstrated that VDSC are important for reaching the highest insulin release rate [105]. A few years later, it was discovered that the VDSC are present in pancreatic β cells from humans and canines and participate in GSIS by depolarizing the membrane of the cells [39,106]. However, their role in rodent pancreatic β cells is intriguing, because VDSC exhibit a voltage dependence of inactivation and, when held at membrane potentials found under physiological conditions, depolarization does not evoke Na^+^ currents in these cells [102,107,108]. 

Despite these important advances in research, we can assume that VDSC have not been as exhaustively studied as other channels in pancreatic β cells have. However, it is well known that VDSC modulators can modify the insulin release ratio [109]. Human pancreatic β cells express Na_v_1.6 and Na_v_1.7 subtypes [39], while Na_v_1.3 and Na_v_1.7 are expressed in rodents [110,111]. Besides, it was found that Na_v_1.7 (the highest expressed) and Na_v_1.3 isoforms significantly contribute to approx. 30% of the current mediated by the Na^+^ channel among the β cell population [111]. Ernest and co-workers (2009) reported that a knockout of Scn1b, which is predominantly expressed in β cells and codes for Na_v_1.7 in pancreatic β cells, causes defects in insulin release [111,112]. Velasco and co-workers (2016) reported that the Na_v_ channels’ classical role in insulin release involves pancreatic β cell activation, soon after closure of the highly glucose-dependent K_ATP_ channel, enhancing depolarization, which promotes Ca^+2^ influx and insulin secretion. Na^+^ influx leads the membrane to further depolarize, since Na^+^ currents activate fast and transiently with a maximum current around −10mV, and this current is completely inactivated milliseconds after it begins. Consequently, high voltage-activated Ca^+2^ channels open [104]. It is widely known that β cells in pancreatic tissue express a different ion channel that uses the electrical signal coupled to blood glucose concentration to determine the insulin release ratio [113,114,115,116], such as Ca^+2^ and K^+^ channels [117,118]. 

Goncalves and co-workers (2003) showed that in the presence of high levels of glucose concentration the activity of VDSC and insulin secretion was enhanced [119]; therefore, it was suggested that their function is related to the variation of glucose concentration [104,119,120]. Years later, Zou and co-workers (2013) proposed that Na^+^ currents may be increased with an increment in glucose levels, which increases ATP production in pancreatic β cells; for this reason, VDSC are connected with the ATP levels or ATP ratio in β cells [121]. The idea is supported by whole cell recordings on pancreatic β cells from slices of rat pancreas, where increased concentrations of ATP from 2 to 8 mM in the recording pipette enhance the activity of the Na^+^ channel. This, in turn, plays a significant role in modulating the cell excitability and insulin secretion in pancreatic β cells, when blood glucose levels increase and change the inactivation curve to more positive potentials, and accelerate the recovery of the inactivation of Na_v_ channels in a dose-dependent manner [121]. In this way, it is possible that the modulation of VDSC by ATP explains their role in glucose-stimulated electrical activity and insulin release, suggesting a metabolic regulation [121]. However, using insulin-secreting cells, Godazcar and co-workers (2018) did not observe an effect on the inactivation of endogenous Na_v_ currents at 5, 11 and 20 mM of glucose, and at acutely increased glucose levels (1 to 20 mM) on Na_v_1.3 and Na_v_1.7 currents they observed no effects on inactivation [122].

It was previously reported that TTX inhibits the Na^+^ current and reduces glucose-stimulated insulin secretion by 55 to 70% [39]. Considering the contradictory and adverse effects in the relationship between the Na^+^ channel and insulin release, further experiments to explore the mechanism of ATP modulation in Na^+^ channel activity are needed. However, it has also been seen that the role of the VDSC in GSIS coupling is far from being a single mediating step between Ca^+2^ entry and inactivation of the K_ATP_ channel; indeed, recent mathematical modeling supports the idea that Na^+^ currents could be more important than previously thought [123]. Usually, VDSC regulate the physiology of pancreatic β cells through mechanisms other than just a depolarizing effect, and there is fascinating evidence that suggests VDSC interact with the metabolism in a bidirectional way. However, the role of these channels remains unknown [58,60,121], and it is still not completely understood in diabetes and metabolic syndrome [26]. Recently, Chen and co-workers (2018) presented the first direct evidence on the role of VDSC in the INS-1 cell line in response to changes in extracellular glucose levels. It was found that as the amplitude of the Na^+^ current decreased, the Na^+^ current of the steady state activation was suppressed with the negative shift in activation curves. Furthermore, the time course of Na^+^ current recovery from inactivation was prolonged, and it increased the insulin release, indicating that these channels of INS-1 cells were involved in the modulation of glucose homeostasis [124]. 

In addition, the activity of VDSC can also be modulated by endogenous signals, which usually act as downstream effectors of signaling cascades, such as the nerve growth factor (NGF), an endogenous molecule that favors pancreatic cell survival and insulin secretion [105,125]; these signals also enhance the activity of Ca^+2^ channels in pancreatic β cells [126]. The effects of NGF on both channel types are mediated by TrkA, a high-affinity receptor with intrinsic tyrosine kinase activity [127].

## 5. Cav Channels and Insulin Secretion

Previous reviews on glucose sensing and insulin release focused mainly on the role of K_ATP_ channels and voltage-dependent calcium channels (VDCCs) and their intrinsic relationship with insulin release, because the closure of K_ATP_ channels opens the VDCCs, allowing the influx of Ca^+2^ through the membrane. In this way, the increment of intracellular calcium acts as the most important messenger for insulin release [45,128,129]. Native pancreatic β cells from several species and also cell lines have different types of VDCCs [19,130], and they are located near insulin granules [128]. It has been seen that the dysfunctions of these channels alter insulin secretion, reinforcing the hypothesis that VDCCs play an important role in GSIS [129]. It has been suggested that Cavβ2 and Cavβ3 subunits could modulate GSIS by the interactions between VGCCs and PKC isozymes [131]. In β cells, the VDCCs mediate the calcium influx through the protein-protein interactions, enzymatic responses and electrical activity, playing an important role in the insulin secretion process; however, VDCCs could also help in the growth, maturation, development, survival and death of β cells [19,126,132,133].

One of the reasons for the biphasic insulin secretion process may reflect the sequential release of different pools of granules, which are associated with different subtypes of L type Ca^+2^ channels [128], and they are best characterized in pancreatic β cells. Since the influx of Ca^+2^ through them represents the largest contribution to increasing [Ca^+2^]_int_, this will favor insulin secretion. Moreover, they are the main contributors to the whole cell Ca_v_ current and insulin granule exocytosis in human, rat, and mouse β cells [16,134]. Pharmacological experiments have shown that 60–80% of the insulin release ratio can be attributed to calcium influx, mainly the L type Ca^+2^ channel pathway [135]. Ca_v_1.2 and Ca_v_1.3 channels (L type) are present in rat, mouse and human β cells. However, the Ca_v_1.3 channel is most expressed in rat and human β cells [134,136]; besides, this channel type is the most important for the insulin secretion process [19,137,138] and plays a role in cAMP homeostasis through adenylate cyclase 1, and it may participate in insulin secretion in an indirect manner [134]. In contrast, the Ca_v_1.2 channel is highly expressed in mouse β cells [19,134,138]. 

The presence of Ca_v_2.1 (P/Q type) and Ca_v_2.3 (R type) channels has been described in β cells from humans and mice, respectively [132] and in all primary and tumoral cell studies to date [129], and these types of channel do not seem to play an important role in GSIS [139]. However, Ca_v_2.3 ablation strongly suppressed the second phase of insulin release [140]. T type Ca_v_3.1 and Ca_v_3.2 channels in rat β cells activate around −40 mV with rapid inactivation and slow deactivation; they may play the role of pacemaker in glucose-stimulating levels [19,104] and in this way play a significant role in the regulation of insulin secretion. 

Several mechanisms, such as Ca^+2^/calmodulin signaling, protein phosphorylation, G protein-coupled receptors and phosphorylated inositol can endogenously regulate Ca^+2^ channels’ activity and their density in pancreatic β cells in the membrane [129]. They all regulate the influx of calcium at survival levels into the cell, and it is important to emphasize that they are among the main mechanisms that regulate L type calcium channels, which are the main focus of this review.

Possible regulation of Ca^+2^ channels and insulin secretion in the pancreatic β cells is regulated by cAMP, PKA, and PKB [129]. An increment of Ca^+2^ influx through VDCCs is observed with cAMP analogs or activators of PKA [141,142]. Besides, mouse β cells showed an increment of exocytotic activity after PKA activation [143], and there was an increment in amplitude in the L type Ca_v_ current in a dose-dependent manner after cAMP-analog exposure [129,144]. PKB interacts with the L type calcium channel activity via phosphorylation of the Cavβ2 subunit, promoting calcium channel trafficking to the membrane and, in this way, PKB participates in GSIS [145]. On the other hand, PKC modulation has been suggested to play a tonic role in maintaining Ca^+2^ channel function, and the effect of Ca_v_ channels by PKC activation is controversial [129]. It is also not clear whether the regulation of Ca^+2^ channels is carried out calcium/calmodulin-dependent kinase II (CaMKII); a reduction of Ca^+2^ influx through Ca_v_ channels and insulin release ratio was observed in pancreatic β cells after CaMKII inhibition [146].

The research of Schwetz and co-workers (2013) showed that activation of somatostatin receptors reduces GSIS in mouse islets by modulating L type calcium channels, and it has been suggested that somatostatin can exert this effect through a Gβγ protein-dependent mechanism [147]. Experiments in the secreting HIT cell line showed an increase in [Ca^+2^]_int_ in response to glucagon, but this effect was blocked by either chelation of extracellular Ca^+2^ or application of Ca_v_1 channel blockers [132,148]. It has been suggested that the effect of GLP-1 on mouse β cells stimulates insulin release due to a slower time-dependent inactivation of Ca_v_ currents; these effects have been seen in Ca^+2^ channels in human and rat pancreatic β cells [132]. Sarah and co-workers suggested that the intracellular II-III loop domain of Ca_v_1.2 and Ca_v_1.3 might target these channels to specific membrane microdomains, allowing their interaction with proteins involved in GLP-1 signaling [149].

Parasympathetic innervation of islets modulates insulin secretion, releasing acetylcholine, which activates muscarinic receptors and stimulates insulin secretion [16], but this acetylcholine also inhibits the Ca_v_ channel activity in mouse β cells [150]. However, studies in HIT cell lines have shown that muscarinic receptors increase the amplitude of whole cell Ca_v_ currents and the probability of the Ca_v_1 channel being open [129]. Nevertheless, little information is available so far on acetylcholine signaling in β cells. Exocytotic proteins like SNAP-25, synaptotagmin and syntaxin1A also interact with L type calcium channels; β cells predominantly express Syn-1A, Syn-2, and Syn-4 in the membrane and Syn-3 in the secretory granules [26]. This interaction modulates the Ca_v_1 channels and the exocytotic activity and could mediate exocytosis of predocked insulin-containing vesicles during the first phase of GSIS [132]. The contributions of the different calcium channel types have not been deeply studied in several species, a reason why this is still controversial.

## 6. Transient Receptor Potential (TRP) Channels and Insulin Secretion 

It is worthwhile considering a different conceptual scenario where TRP channels could possibly be involved in the regulation of the insulin secretion process. TRP channels are composed of non-selective cation channels that act as cellular sensors and mediate the responses to changes in the extracellular environment [151]. They also play an important role in the background current that contributes to membrane depolarization after K_ATP_ channels’ closure [152] and in cellular processes such as hormone secretion and reloading Ca^+2^ stores [151]. TRP channels are present in pancreatic β cells (human, mouse, rat) and insulin-secreting cell lines [151,152,153], and they have been suggested to be involved in the regulation of [Ca^+2^]_int_ [154]. In mammals, TRP channels are grouped in six sub families [155], and almost all of them are located on the intracellular membranes, in addition to the plasma membrane [156]; all share a common tetrameric structure, which is similar to the voltage-gated K^+^ channel [157]. TRP channel function is influenced by large intracellular domains that can be modulated by phosphoinositides, ammonium ions and venom toxins [158,159,160].

TRPM2 channels could contribute to insulin release induced by glucose, temperature stimuli and incretin hormones [161,162]; for example, exedin-4 is a GLP-1 receptor agonist that favors the insulin release from rat pancreatic β cells. In islets after shRNA-mediated knockdown of TRPM2 expression, the insulin release was significantly reduced [161], and it has been described in pancreatic β cells that the Ca^+2^ entry through TRPM2 channels acts as an inductor of GSIS after protein kinase A activation [162,163]. TRPM5 and TRPM4 channels are candidates for inducing cellular depolarization in response to increasing [Ca^+2^]_int_ concentration. It has been shown that TRPM5 channels have a functional role in pancreatic β cells, and this function is involved in the regulation of the insulin release process [164]. Recently, TRMP5 channels have been considered as a modulator of GLP-1R in a PKC-dependent pathway; and these channels are shown to be at least partly responsible for the GLP-1 mediated insulinotropic effects [165]. The pharmacological potential of TRPM5 should be to lead to the stimulation of the physiological path in the insulin secretion process [164,166]. On the other hand, the TRPM5 channel has been considered as an important determinant of Ca^+2^ oscillation in mouse β cells [164], and the absence of this channel causes significant glucose intolerance in adult animals [164,167]. In addition, TRPV1 channels might also be an interesting drug target for compounds that modulate channel activity, insulin release and GLP-1 secretion [168]. The depolarization caused by the activation of TRPM3 channels could lead to the activation of L type Ca^+2^ channels [169,170], while activators of these channels also enhance glucose-dependent insulin release [170,171]. 

TRPM4 channels are a component in the control of [Ca^+2^]_int_ signals for insulin release, and it is probable that TRPM 4 channels are not involved in the signal mechanism following glucose stimulation, but this does not exclude a possible role of TRP4 channels in G-receptor (Gq-or Gs receptor-coupled) signaling pathways, as has been seen during stimulation with glucagon. Besides, GLP-1 insulin release stimulation is at least partially dependent on PKC and TRMP4 channel activation [165]. The TRPV4 channel is expressed in mouse pancreatic islets and min6 β cells, where it confers a human islet amyloid polypeptide (hIAPP)-induced rise of the intracellular Ca^+2^ levels that activated these channels; however, hIAPP induces cytotoxicity and contributes to the loss of β cells [172]. Recently, Skrzypski et al., 2013 showed that TRPV4 triggers glucose-stimulated insulin secretion by modulating intracellular calcium [173].

Evidence suggested that several TRP channels regulate the insulin secretion process and pancreatic β cells function through the ability of these channels to increase the [Ca^+2^]_int_ and membrane depolarization, which could be one of the functions that they play in GSIS in the pancreatic β cells [174], but their specific role in the insulin secretion process has not been fully unraveled [175]. In mammals, it has been seen that the participation of these channels in metabolic status and particularly in the pathophysiology of pancreatic β cells has increase over the years. There is no doubt of the importance of these channels in controlling insulin secretion in response to many stimuli [163,176]. Considering the side effects of sulfonylureas, it is necessary to develop new insulin secretagogues, and TRP channels are interesting targets for this development. Indeed, several TRP channels have an important potential for use as a target for insulin-tropic drugs, a feature that makes the investigation of their function in human islets an urgent and challenging task.

## 7. K_v_ Channels and Insulin Secretion

The insulin secretion process ends when the pancreatic β cells are repolarized by the activation of potassium channels such as K_v_ and KCa [39,40,41]. It has been recognized that voltage-dependent outward K^+^ currents in insulin-secreting cells are involved in both K_v_ and KCa current components, where the K_v_ component contributes 80 to 85% of total voltage-dependent outward currents, while the KCa component contributes 15 to 20% [177,178,179]. Although the role of K_v_ channels involved in the regulation of GSIS still remains obscure [180], recent research has suggested that K_v_1, K_v_2 and KCa channels, and recently K_v_1.7 channels, are important for the insulin secretion process.

It has been suggested that K_v_1 channels represent 25% of the β cells’ delayed rectifier currents, whereas K_v_2 channels represent 60% [181,182,183,184], and the latter are considered the most prominent K_v_ channels in β cells [152]. K_v_2.1 channels serve as a break for glucose-stimulated insulin secretion [152,177,184,185], and their inhibition enhances GSIS [181,182,183,184]. These channels activate gradually and inactivate slowly or do not inactivate at all, and they mediate the majority of the voltage-dependent outward K^+^ current in mouse and rat β cells [152,177,184]. Several studies identify the K_v_2.1 channel as the major contributor to voltage-dependent outward K^+^ currents in rodent pancreatic β cells and insulinoma cells lines [177,184] and regulate excitability [Ca^+2^]_int_ and insulin secretion [177,184]. The K_v_2.1 channel has been reported to be involved in the maintenance of fasting blood sugar during the burst of β cells’ insulin release between meals, but it is considered a difficult pharmacological target due to its widespread expression [186]. 

KCa channels are regulated commonly by intracellular Ca^+2^ [19], and the kinetics of the currents suggests the capability of these channels to modulate electrical activity, including action potential shape and amplitude [40,41]. The BK type is present in pancreatic β cells, and their important role in GSIS was evident when blockage of these channels increased action potential amplitude and enhanced the insulin release by 70%; in contrast, the inhibition of K_v_2.1/2.2 channels’ activity did not show any stimulatory effects on electrical activity and insulin secretion [39]. It has also been suggested that the BK type participates in repolarizing the plateau or the spikes [187], as does the K_v_2.1 channel [186]. The expression of K_v_1.7 at high levels in rodent islets suggests that these K_v_ subtypes to the remainder of the β cells delayed rectifier current [178,188], and it was also elucidated that the gene for human K_v_1.7 mapped to chromosome 19q13.3; it is a region considered an important diabetes susceptibility locus [189]. Finol-Urdaneta and co-workers (2012) described a toxin (Conkunitzin-S1) that blocks the K_v_1.7 channel subtype, and as a consequence there is an increasing glucose-stimulated insulin release due to the increase in action potential firing [180]. They thus demonstrated that these channels are physiologically relevant for the pancreatic insulin secretion process, and indicated that K_v_1.7 activity contributes actively to the control of GSIS in pancreatic β cells [180].

Pharmacological inhibition and even genetic ablation of K_v_ channels result in a prolongation of action potential duration that increases [Ca^+2^] and insulin secretion [177,186,190], which has been seen in mouse β cells in the presence of antagonist tetraethylammonium (TEA) [191,192]. It is important to emphasize that inhibition of K_v_ channels results in potentiation of the insulin secretion process in a glucose-dependent manner, because in physiological conditions these channels only open in response to membrane depolarization at high glucose level [185]. Previous studies have shown that the activation of Ca^+2^ channels and mobilization of calcium from intracellular stores are responsible for cAMP-regulated cellular function [144,193]. Liu and co-workers (2017) provided new evidence that demonstrates the ability of cAMP signaling to enhance [Ca^+2^]_int_, and insulin secretion is also due to the inhibition of K_v_ channels; it is suggested that the cAMP/K_v_ channel pathway could be a therapeutic strategy for diabetes treatment [194]. This point of view is also supported by the studies with the GLP-1 receptor agonist that potentiated insulin secretion in a glucose-dependent manner through the cAMP pathway [195,196]. Since pancreatic β cells’ K_v_ current is considered a potent glucose-dependent regulator of insulin secretion, MacDonald & Wheeler hypothesized that the physiological secretagogue GLP-1 could regulate K_v_ channel function. In general, the glucose dependence of the insulinotropic effect of K_v_ inhibitors makes these channels promising targets for the development of treatments [190]. 

Finally, ERG K^+^ channels are also part of the larger family of K_v_ channels [197]. They are present in pancreatic α and β cells [198,199] and modulate cellular activity by controlling the repolarization of the action potential [200,201]. It has been suggested that inhibition of Erg1 K^+^ channel activity in β cells enhances insulin secretion under high glucose-stimulatory conditions, while in the alpha cell, channel blockage inhibits secretion of glucagon under low glucose conditions [198]. This could be a possible therapeutic mechanism for the treatment of diabetes.

## 8. Calcium as a Messenger for Insulin Secretion

The electrophysiological properties of any given cell are determined by its ion channel activity. The manner in which the different ion channels contribute to electrical activity in mouse and human pancreatic β cells has been briefly outlined; all of them favor the increment of [Ca^+2^]_int_. Ca^+2^ is an important messenger for diverse biological activities [202], and changes in its concentration in the different compartments on the cell are crucial [203]. Thorough studies have shown that intracellular Ca^+2^ levels in pancreatic β cells are maintained by Ca^+2^ entry, predominantly through VDCC, Ca^+2^ uptake and release from organelles and intracellular stores, and the Ca^+2^ expulsion via plasma membrane pumps such as Ca^+2^ ATPase (PMCA) and exchangers such as the Na^+^/Ca^+2^ exchanger (NCX) [204,205,206], so any alterations in this regulatory process can affect the insulin secretion process [207].

Na^+^/Ca^+2^ exchange represents an important modulator of cytosolic free Ca^2+^ concentration and participates in both Ca^2+^ outflow and Ca^2+^ inflow, depending on the state of cell activity [208]. This exchange can also be stimulated by glucose and membrane depolarization [209]. For this reason, the regulation of Na^+^/Ca^+2^ exchange may be of great interest in the understanding of the stimulus for secretion coupling of glucose-induced insulin release from the pancreatic β cells; however, it must be kept in mind that Na^+^/Ca^2+^exchange is a complex system that allows both Ca^2+^ entry and outflow and generates, in addition, an inward current, while the plasma membrane PMCA only extrudes Ca^2+^ [210].

As seen in rat pancreatic β cells, Na^+^/Ca^2+^exchange displays quite a high capacity [209] and participates in the control of [Ca^2+^]_int_ and insulin release [211,212]. Van Eylen and co-workers confirmed in 2002 that Na^+^/Ca^2+^exchange plays an important role in Ca^2+^ homeostasis, suggesting that the current generated by this exchange shapes stimulus-induced membrane potential and [Ca^2+^]_int_ oscillations in insulin-secreting cells [210]. It is important to mention that in the exocytosis of insulin granules in pancreatic β cells, the cyclic AMP (cAMP) is the most important regulator and amplifier besides calcium [213,214,215]. The cAMP promotes secretion of insulin at several levels, while the related mechanisms that have been proposed include mobilization of Ca^+2^ from intracellular stores [216,217] and modulation of ion channel activities from K_ATP_ channels [218], L type voltage-dependent Ca^+2^ channels [219] and the non-selective cation channels [220]. This may take place either by increasing the electrical activity, [Ca^2+^]_int,_ by recruiting granules or by acting directly on the exocytosis machinery that accounts for as much as 80% of its effect [217]. This is the reason why cAMP has the most important effects on secretory granule trafficking and exocytosis [150] and also can be the second messenger in the insulin release process. In this way, Ca^+2^/cAMP signaling interaction could be a new therapeutic target for antidiabetic medicines [221] and other diseases [222]. 

It has been established that inhibition of K_v_ channels boosts insulin secretion via prolongation of action potential [152,186], but there is also evidence suggesting that endogenous or exogenous enhancement of cAMP profoundly inhibits K_v_ channels, which in turn prolongs action potential and increases [Ca^+2^]_int,_ suggesting that targeting the cAMP/K_v_ channel pathway may be a therapeutic strategy for diabetes treatments [194]. Finally, the effects of cAMP on pancreatic β cells are not restricted to regulation of membrane potential or the influx of calcium, but cAMP can also be involved in the mobilization of the ion from intracellular stores and other different signaling (insulin and glucagon) on the cells (more information see review [223].

## 9. Sulfonylureas and Diabetes 

Sulfonylureas have been the group of drugs used as the main treatment of T2D for a long time, and they act in a glucose-independent manner [184,224,225]. In 1995, before the cloning of the SUR 1 subunit was carried out, the high affinity sites for sulfonylureas were localized in the membrane of β cells [226,227] with a dissociation constant at nanomolar range [47], which was the molecular target of these compounds [81]; moreover, their adverse effects were also improved [225,228,229]. Sulfonylureas induced the closure of K_ATP_ channels and depolarization of the membrane, leading to the opening of calcium channels favoring ion flow into the cell, generating insulin release by β cells in the pancreas [45]. As a result, they have been used as oral hypoglycemic agents for more than 50 years [225]. Nonetheless, clinically, these inhibitor compounds are used to treat T2D and hypoglycemic states, respectively [230,231,232,233]. In this scenario, it is important to find other strategies for treatment of disease, and natural resources such as toxins from animal venom could be a good option.

## 10. Natural Resources and Diabetes

Animal venoms are unique sources of compounds that target receptors and ion channels [234,235,236], and over the past 80 years toxins found in venomous and poisonous animals have been studied in order to understand the biochemical and physiological mechanisms by which these toxins lead to pathological consequences; it was also found that there are a few toxins with therapeutic use [235]. A very brief summary is provided here of toxins isolated from venomous and poisonous animals from which therapeutic drugs have been developed or are under development, and other toxins that could have potential therapeutic use in favoring the insulin secretion process (Table 1).

## 11. Antidiabetic Agent from Lizard Venom: Exenaide

Between 2000 and 2013, 1453 new drugs were approved by the US Food and Drug Administration (FDA) [280], but despite the number of studies using compounds isolated from animal venoms with high selectivity and specificity for different molecular targets [281], the successful cases in the development of new therapeutic drugs are still rare [282]. An interesting example is exenaide, the synthetic version of exendin-4 (GLP-1), with 39 amino acid residues isolated from *Heloderma suspectum* venom. It shares 53% homology with the human GLP-1 [237] and avidly binds to the GLP-1 receptor on the surface of pancreatic β cells [283]; it is largely resistant to the action of serine protease Dipeptidyl peptidase-4 (DPP-4) [284]. The greater stability of the peptide exendin-4 sparked its rapid development and it is currently used for the treatment of T2D, because this compound reduces both fasting and prandial glucose levels [285]. In addition, it has been demonstrated that this compound limits food intake and increases satiety in normal glycemic and hyperglycemic individuals, with a consequent reduction in body weight [286,287,288]. It significantly increases the two phases of insulin secretion process in patients with T2D, indicating an improvement in the response of pancreatic β cells [289]. Exedin-4, a venom peptide, provides the first example for therapeutic application for metabolic diseases such as diabetes, and expert opinion positioned it as an effective and safe drug for the treatment of T2D that has been available as exenatide (Byetta^®^) since 2005, albeit presenting nausea as an adverse effect [290,291].

## 12. Peptides with Potential Utility in the Development of New Diabetes Therapeutics

In addition to exendin-4, other Glp-1 analogues from *Ornithorhynchus anatinus* (platypus) and *Tachyglossus aculeatus* (echidna) could be good candidates for T2D [292]. These peptides show interesting biophysical characteristics, including resistance to DPP-4 cleavage in a similar way to exendin-4, at concentration of 100 nM, equivalent stimulation of the insulin secretion process and relative bias toward pERK1/2, which is involved in the activation of mitogenic signaling pathways, unlike human GLP-1 and exendin-4 with relative bias for cAMP and intracellular calcium mobilization [244]. These signaling differences generated by monotreme peptides could be another option for the development of new GLP-1 as anti-diabetic agents, such as several peptides and their analogs isolated from skin of frog, especially, the insulinotropic peptide “FSIP” isolated from the skin secretion of the frog *Agalychnis litodryas*, which is in phase 3 of clinical trials [293]. 

Most of the natural peptides that help in the insulin secretion process involve the GLP-1 pathway, and just a few interact with ionic channels. This is the reason why incretin-based therapies are becoming increasingly important in the treatment of patients with T2D [294]. In the pancreatic β cells, the insulin secretion process involves several stimuli, which makes this biological activity a complex process, but it is glucose that plays the major role [16]. In this context, toxins like guangxitoxin-1 and α-latrotoxin enhance insulin secretion in a glucose-dependent manner [182,272,273]. On the other hand, the toxin CTX-I isolated from *Naja kaouthia* snake venom stimulated insulin secretion in a concentration-dependent manner in the absence of glucose [249]; this type of insulin secretion modulator is also highly attractive for the treatment of T2D [109]. 

## 13. Other Options: Peptides-ionic Channel Interaction 

Blocking of K_ATP_ channels is the principal target of Sulfonylureas and, in natural resources, a new inhibitor peptide (SpTx-1) was recently isolated from *Scolopendra polymorpha* (spider pharm) venom with high affinity to the human ATP-sensitive K_IR_6.2 channel [295]. Ramu and co-workers (2018) show that SpTx-1 inhibits the K_ATP_ channels from the extracellular side with a dissociation constant value of 15 nM in a relatively specific manner and in an apparent one-to-one stoichiometry [295]. This is the first evidence for a natural peptide that inhibits the channel by primarily targeting the K_IR_6.2 subunit rather than the SUR 1 receptor. This peptide could be an effective tool for new discoveries in the physiological roles of K_ATP_ channels and might be an appropriate target for diabetes treatment, especially neonatal diabetes mellitus, which is insensitive to sulphonylureas. However, there is still no evidence that SpTx-1 favors the insulin secretion process. On the other hand, the peptide mastoparan has been suggested to interact with these channels as well, but it has also been evaluated in different biological responses, to define the molecular mechanism involved in insulin secretion [274,275,276,277,278,279] (Table 1). Recently, Moreno and Gilart 2015 described mastoparan as toxic with a wide variety of biological effects [296], suggesting different biological applications for the peptide, but they did not consider insulin release, and maybe this is due to the lack of specificity of the peptide for this particular biological activity.

Amphibian skin secretions are a rich source of peptides with pharmacological properties that show potential for the development of antidiabetic agents [297]. A number of peptides and their analogs have been described, which favor the insulin secretion process by different pathways [297], such as GLP-1 receptors [252,253,255,256,257], K_ATP_ channel blocking in BRIN-BD11 cells [298], K_ATP_ channel-independent pathways [250], K_ATP_ channel-dependent pathways [299], or Ca^2+^-independent pathways [251] accompanied by physiological effects such as membrane depolarization and increased Ca^2+^ concentration [255,269,300,301,302]. Further, some of these peptides can interact with cAMP protein kinase A and C dependent G-protein sensitive pathways [258]. Basically, these amphibian peptides involve the most important signaling pathways for insulin release in pancreatic β cells, and most of them act without compromising the integrity of the plasma membrane; for this reason, this kind of peptide could be as important as the antimicrobial peptides (Table 1). Besides, there is evidence about several frog peptides at different concentrations (nM) that significantly and modestly enhance insulin release such as amolopin [303]; palustrin-1c [304]; xenopsin and xenopsin-AM2 [257]; plasticin-L1 and ocellatin-L2 [305]; phylloseptin L2 [251]; ranatuerin-2CBd, palustrin-2CBa, temporin-CBa, temporin-CBf [306]; brevinin-2GUb [307]; temporin-ITa [308], but unfortunately the mechanism is unknown.

In snake venoms several fractions have been found that significantly increase insulin release and do not exhibit any cytotoxic effects. For example, seven fractions isolated from *Bitis nasicornis* and *Crotalus vegrandis* snake venom that interfere in cell signaling pathways activated by integrins, such as receptor tyrosine kinases, may impact positively on insulin secretion [309]. However, the complete sequences of these fractions are not available yet. Crotoxin (crotapotin and PLA_2_ subunits) isolated from *Crotalus durissus collilineatus* snake venom stimulated secretion and is not dependent on an additional influx of Ca^2+^ through L-type channels, but instead is associated with arachidonic acid formation in pancreatic islets. The integrity of the plasma membrane of the pancreatic β cells was not altered by the exposure of the islets to PLA_2_, which is responsible for the secretion process [310]. 

For many years, modulators or blockers of K_ATP_ channels have been used for T2D, and research continues in this area. However, in recent years, the evidence has demonstrated that blockage or modulation of other potassium channel types such as K_v_1.3, K_v_1.4, K_v_1.7 K_v_2.1, K_v_2.2 and the BK channel could be an attractive target for the management of T2D [39,181,182,183,184,190]. At the moment, only four peptides isolated from spiders, scorpion and conus (Table 1) block the potassium channel and increase insulin secretion; therefore, these toxins show another possible therapeutic pathway by which to treat diabetes [292], besides K_ATP_ channels. In this way, this anti-diabetic pathway could be more direct and focused whenever these toxins have a specific target in specific tissue and without secondary adverse effects.

Despite extensive research, selective channel blockers are limited, and most toxins that interact in these channels are rather non-selective [311]. For example, pharmacological inhibitors of the BK channel provoke an increase in the myogenic tone of various arteries [312]. Otherwise, it is important to keep in mind that the small size, compact and rigid structure, high potency, and selectivity of peptide inhibitors of mammalian potassium channels, such as charybdotoxin, margatoxin, and maurotoxin [313], have become valuable tools for research and drug development, including for diabetes. Toxins that modulate sodium channels, such as TsTx-V, depolarize mouse pancreatic β cells, increasing insulin secretion twofold over basal values [119], but the exact mechanism of action is unknown. 

## 14. Conclusions

Diabetes is one of the most important diseases in the world, and it requires special attention. In recent decades, several treatments have been successfully used, but adverse effects are unfortunately an issue. In this context, the search for new treatments is urgent and challenging, and the toxins present in animal venoms may be an important source. The selectivity of some toxins for special targets such as ion channels has made them good candidates as molecular tools in the treatment of different diseases. We have outlined the toxins found in different groups of animals that contribute to the insulin secretion process; most of them are at the stage of simple basic research, one is currently under clinical trials or being developed for eventual therapeutic use, and one of them is a drug for diabetes treatment. In this line, a very important discovery for diabetes treatment was the peptide exendin-4, isolated from the venom in the saliva of the lizard *Heloderma suspectum*. It is now the only compound from natural sources being used to treat diabetes with success, but other insulinotropic peptides isolated from *Agalychnis litodryas, Ornithorhynchus anatinus* and *Tachyglossus aculeatus* could be on their way to becoming drugs. At the moment, we can see that most of the peptides from natural sources that help in the insulin secretion process involve the GLP-1 pathway, and just a few interact with ionic channels. Therefore, in this review, we aimed to provide a better understanding of the ionic mechanisms involved in the insulin secretion process and the importance of the use of natural resources for the characterization or treatment of several illnesses such as diabetes, highlighting the ionic channel as an alternative pathway for treatment. However, it is also important to emphasize the toxins that modulate insulin secretion, even though there have not been significant advances in the search to clarify their modulating role, because with the information available, suggestions only can be made. 

## Figures and Tables

**Table 1:** Insulin release mechanism modulated by toxins isolated from animal venoms.

Mode of Action	Toxins	Species	Sequence	aa	Summary	Ref.
**Specific Mechanism**						
GLP-1 receptor Agonist	Exendin-4 (Exenatide; Byetta ™)	*Heloderma Suspectum*	HGEGTFTSDLSKQMEEEAVRLFIEWLKNGGPSSGAPPPS	39	Highly stable GLP-1 analogue, promotes glucose-dependent insulin secretion, inhibits glucagon secretion and suppresses appetite; Increase in cAMP signaling in pancreatic acinar cells with superior selectivity; Ex-4 may stimulate β-cells cooperatively by inhibiting K_ATP_ channels and activating TRPM2 channels (potential target for T2D); Human GLP-1 and ex-4 show relative bias for cAMP and intracellular Ca2 mobilization (involved in promotion of insulin release)	[237,238,239,240,241,242,243]
	Platypus GLP-1 (pGLP-1)	*Ornithorhynchus Anatinus*	HSEGTFTNDVTRLLEEKATSEFIAWLLKGLE	31	Stable GLP-1 analogue with distinct signal bias; stimulates insulin release in cultured rodent islets; Resistance to DPP-4 cleavage; Biophysical characteristics that may offer valuable insight in the development of new anti-diabetic agents	[244]
	Echidna GLP-1 (eGLP-1)	*Tachyglossus Aculeatus*	HFDGVYTDYFSRYLEEKATNEFIDWLLKGQE	31		
Insulinotropic peptide	FSIP	*Agalychnis litodryas*	AVWKDFLKNIGKAAGKAVLNSVTDMVNE	28	Clinical phase 3; Type 2 diabetes	[245]
Modulate Na^+^ channel inactivation.	TsTx-V	*Tityus serrulatus*	KKDGYPVEGDNCAFACFGYDNAYCDKUIKDKKADDGYCYWSPDCYCYGLPEHILKEPTKTSGRC	64	Membrane depolarization and increased relative duration of electrical activity during the active phase.	[119]
	Crotamine Two isoforms (F2 – F3)	*Crotalus durissus terrificus*	YKQCHKKGGHCFPKEKICLPPSSDFGKMDCRWRWKCCKKGSG	42	Increases the peak of Na^+^ currents and inhibits the direct transition of the channel from closed to inactivated states; Insulin secretion in dose-dependent manner.	[246]
	Cro 2	*Crotalus durissus cascavella*	YKRCHKKGGHCFPKEKICLPPSSDLGKMDCRWKRKCCKKGSGK.	43	Cro 2 binding to open state of the Na^+^ channels, induces a dose-dependent manner of insulin secretion; The most important region for the binding of Cro 2 to the Na^+^ channels involved the C-terminal region.	[247]
BK channel blocker	Iberiotoxin (IbTx)	*Buthus tamulus*	ZFTDVDCSVSKECWSVCKDLFGVDRGKCMGKKCRCYQ	37	Increases action potential amplitude, enhances insulin secretion by 70% and increases resting membrane conductance.	[39]
K_v_2.1 channel blocker	Hanatoxin (HaTX)	*Grammostola rosea*	ECRYLFGGCKTTSDCCKHLGCKFRDKYCAWDFTFS	35	Inhibits the delayed rectifier current by 65% at +20 mV; Induces a right shift in the current-voltage (*I*-*V*) relationship; Affects oscillatory [Ca_2_]_int_ responses in mouse and human islets in the presence of glucose.	[181,248]
K_v_2.1 and K_v_2.2 channel blocker (β cell IDR )	Guangxitoxin-1 (GxTX-1)	*Chilobrachys Guangxiensis*	EGECGGFWWKCGSGKPACCPKYVCSPKWGLCNFPMP	36	Inhibits 90% of the β cell IDR; Increases [Ca_2_]_int_ and stimulated insulin secretion in a glucose-dependent manner.	[182]
K_V_1.7 channel blocker	Conkunitzin-S1 (Conk-S1)	*Striated cone*	KDRPSLCDLPADSGSGTKAEKRIYYNSARKQCLRFDYTGQGGNENNFRRTYDCQRTCLYT (amidation of the C-terminal)	60	Conk-S1 as a specific blocker of K_v_1.7 and indicates that K_v_1.7 activity contributes actively to the control of GSIS in pancreatic β cells without affecting basal glucose;	[180]
**Non-specific Mechanism**						
K^+^ channel Blocker (by similarity)	Cardiotoxin-I (CTX-I)	*Naja kaouthia*	LKCNKLIPIASKTCPAGKNLCYKMFMMSDLTIPVKRGCIDVCPKNSLLVKYVCCNTDRCN	60	Stimulates insulin secretion in a concentration-dependent manner in the absence and presence of glucose without affecting cell viability and integrity; Increases the mobilization of [Ca_2_]_int_; Insulinotropic activity does not involve GLP-1R signaling.	[249]
K_ATP_ channel independent pathway	Temporin-1Oe	*R. ornativentris*	ILPLLGNLLNGLL.NH2	13	Stimulates in vitro insulin release from BRIN-BD11 rat insulinoma derived cells; does not stimulate release of the cytosolic enzyme LDH.	[250]
	Temporin-1Va Temporin-1Vc Temporin-1Vb	*R. virgatipes*	FLSSIGKILGNLL.NH2 FLPLVTMLLGKLF.NH2 FLSIIAKVLGSLF.NH2	13	Small but significant increases in insulin release with no increased rate of LDH.	[250]
	Temporin-1DRb	*R. draytonii*	NFLGTLVNLAKKIL.NH2	14	Stimulates in vitro insulin release from BRIN-BD11 rat insulinoma-derived cells; does not stimulate release of the cytosolic enzyme LDH.	[250]
	Temporin-1TGb	*R. tagoi*	AVDLAKIANKVLSSLF.NH2	16	Stimulates in vitro insulin release from BRIN-BD11 rat insulinoma derived cells; does not stimulate release of the cytosolic enzyme LDH.	[250]
Ca2+-independent pathways	Pseudin-2	*Pseudis paradoxa*	GLNALKKVFQGIHEAIKLINNHVQ	24	Stimulates insulin release from BRIN-BD11.	[251]
GLP-1 receptor Agonist	RK-13	*Agalychnis calcarifer*	RRKPLFPLIPRPK	13	Stimulated insulin release in a dose-dependent, glucose-sensitive manner, exerting its effects through a cyclic AMP-protein kinase A pathway independent of pertussis toxin-sensitive G proteins.	[252]
GLP-1 receptor Agonist	Brevinin-2-Related Peptide	*Lithobates septentrionals*	GIWDTIKSMGKVFAGKILQNL.NH2	21	The rate of insulin release increased to 222 % of basal rate.	[253]
	Magainin-AM1 Magainin-AM2	*Xenopus amieti*	GIKEFAHSLGKFGKAFLGEIMKS GVSKILHSAGKFGKAFLGEIMKS	23	All peptides produced a significant increase over the basal rate of insulin release; Magainin-AM2 was the most potent peptide and Magainin-AM1 stimulated GLP-1 release.	[254,255]
	Hymenochirin–1B	*Hymenochirus* *boettgeri*	IKLSPETKDNLKKVLKGAIKGAIAVAKMV.NH2	29	This peptide produced a concentration-dependent increase in the rate of insulin release from BRIN-BD11 cells without cytotoxicity	[256]
	Caerulein-B1	*Xenopus borealis*	EQDY(SO3)GTGWMDF	11	At concentrations ≥30 nM stimulates the rate of insulin secretion from BRIN-BD11 cells with a maximum response (360% of basal rate) at 3 µM	[257]
	PGLa-AM1 PGLa-AM2 XPF-AM1 CPF-AM1 CPF-AM3 CPF-AM4 CPF-AM2	*Xenopus amieti*	GMASKAGSVLGKVAKVALKAAL.NH2 GMASTAGSVLGKLAKAVAIGAL.NH2 GWASKIAQTLGKMAKVGLQELIQPK GLGSVLGKALKIGANLL.NH2 GIGSALAKAAKLVAGIV.NH2 GLGSVLGKILKMGANLLGGAPKGA GLGSLVGNALRIGAKLL.NH2	22 22 25 17 17 24 17	All peptides produced a significant (*p* < 0.05) increase over the basal rate of release at concentrations P300 nM. Magainin-AM2 was the most potent peptide, producing a significant (*p* < 0.05) increase at a concentration of 1 nM. CPF-AM1 was the most effective peptide, producing a maximum response 3.2-fold greater than basal rate (*p* < 0.001) at 3 µM concentration	[254,255]
G protein interaction	Esculentin-1b	*Pelophylax saharicus*	GIFSKLAGKKLKNLLISGLKNVGKEVGMDVVRTGIDIAGCKIKGEC	46	Stimulates insulin release from rat RINm5F insulinoma-derived cells and increases intracellular Ca2+ concentrations	[258]
	Agelaia MP-I (AMP-I)	*Agelaia pallipes pallipes*	INWLKLGKAIIDAL–NH2	14	Increases glucose-induced insulin secretion in a dose-dependent manner and this effect was not due to lysis process; The mechanism involved in this modulation is independent of the K_ATP_ and L-type Ca^2+^ channels, suggesting a different mechanism for this peptide, possibly by a G protein interaction.	[259]
Insulin receptor agonist	Insulin 1 (Con- Ins G1)	*Conus geographus*	GVVgHCCHRPCSNAEFKKYC (A chain) TFDTOKHRCSGSgITNSYMDLCYR (B chain)	44	Con-Ins G1 the smallest insulin identified from a natural source; Injection of Con-Ins G1 into zebrafish produced a rapid drop in blood glucose with a potency comparable to that of human insulin; Con-Ins G1 could be an effective drug for T1D.	[260,261]
Phospholipase A_2_ (PLA_2_) arachidonic acid release	BcPLA_2_1	*Bunodosoma caissarum*	GATIMPGTLWCGKGNSAADYLQLGVWKDTAHCCRDHDGC	39	Strongly induces insulin secretion only in presence of high glucose concentration; The enzymatic activity of BcPLA_2_1 is not required for its pharmacological activity.	[262]
	Melittin	*Apis mellifera*	GIGAVLKVLTTGLPALISWIKRKRQQ	26	Stimulates insulin secretion in isolated rat islets in a dose-dependent manner by activating phospholipase A2 in islet cells, causing release of arachidonic acid from membrane phospholipid; The effect on insulin secretion was dependent on extracellular calcium and did not require the presence of glucose.	[263,264,265]
	BjVIII Lys49-PLA2 phospholipase A2 (sPLA2) isoform	*Bothrops jararacussu*	SLFELGKMILQETGKNPAKSYGAYGCNCGVLGRGGPKDATDRCCYVHKCCYKKVTGCDPKKDRYSYSWKDKTIVCGENNPCLKELCECDKAVAICLRENL GTYNKKYRYHLKPFCKKADPC ^38^DATDRCCYVHK^48^ new isoform Lys49-PLA2 ^20^SYGAYGCNCGVLRGGPK^36^, the calcium binding region	121	Enhances insulin secretion by increasing calcium entry into pancreatic β cells; hyperactivating depolarization-induced exocytosis; does not show significant enzymatic activity; Arachidonic acid induces an increase in insulin secretion, which may be due to potential interaction with K+ channels and the stimulation of adenylyl cyclase guanydyl cyclase, proteinkinase C (PKC),the Ca2+/calmodulin-dependent protein kinase; inhibition of K_ATP_ channels	[266]
Phospholipase A_2_ (PLA_2_) arachidonic acid release	Phospholipase A2	*Naja mossambica mossambica*	NLYQFKNMIHCTVPSRPWWHFADYGCYCGRGGKGTPVDDLDRCCQVHDNCYEKAGKMGCWPYFTLYKYKCSQGKLTCSGGNSKCGAAVCNCDLVAANCFAGARYIDANYNINFKKRCQ	118	Induces insulin secretion also involved in block of ATP-dependent K channels, increases cytosolic free Ca^2+^ and insulin exocytosis; Despite the differing opinions on the mechanism of action of PLA2-induced insulin secretion in β cells, its role as a potent insulin secretagogue is without question.	[267]
Mechanism involving membrane depolarization and increase of intracellular Ca2+	Alyteserin-2a	*Alytes. obstetricans*	ILGKLLSTAAGLLSNL.NH2	16	Significant stimulation of the rate of insulin release from BRIN-BD11.	[268]
	Esculentin-2CHa	*Lithobates chiricahuensis*	GFSSIFRGVAKFASKGLGKDLAKLGVDLVACKISKQC	37	Stimulates insulin secretion from rat BRIN-BD11 clonal pancreatic β cells	[269]
PTK-dependent pathway	Convulxin-Like Protein (Cvx-like)	*Crotalus durissus collilineatus*	α subunit: GLHCPSDWYAYDGHCYKIFNEEMNWED β subunit: GFCCPSHWSSYSRYCYKFFSQEMNWEDAEK	57	Insulin secretion induced by Cvx-like protein may be mediated by a protein tyrosine kinase-dependent pathway and may involve other membrane receptors, such as GP VI or Scr family proteins.	[270]
Exocytosis process mediated by receptor	α-Latrotoxin (α -LTX)	*Latrodectus tredecimguttatus*	Polypeptide: 21-1199 aa		*α*-LTX receptors are expressed on primary β cells and the toxin induces exocytosis of the peptide hormone insulin in a glucose-dependent manner in both the presence and absence of extracellular Ca^2^^+^; rises in cytosolic Ca^2^^+^, large conductance of non-selective cation channels; and Ca^2+^ dependent insulin granule exocytosis; α-LTX induces signaling distinct from pore formation via full-length LPH and phospholipase C to regulate physiologically important K+ and Ca^2^^+^ channels as novel targets of its secretory activity.	[271,272,273]
Insulinotropic toxin; GTP-binding protein; Exocytosis process; K_ATP_ Channel inhibition; Direct activation of Rho protein	Mastoparan Versatile peptide	*Vespula lewisii*	INLKALAALAKKIL	14	Mastoparan, an amphiphilic tetradecapeptide, inserts itself into the phospholipid bilayer of the plasma membrane and displays four positive charges near the inner surface of the membrane. This structurally mimics the positively charged loops of hormone receptors that associate with the R-subunit of heterotrimeric GTP-binding proteins; Mastoparan suppresses K_ATP_ channel activity and causes depolarization, implicating this pathway in mastoparan-induced [Ca^2+^]ı elevation; Mastoparan had no effect on cellular _C_AMP levels.	[274,275,276,277,278,279]

aa: amino acid residue.
